# Measuring satisfaction with health care services for Vietnamese patients with cardiovascular diseases

**DOI:** 10.1371/journal.pone.0235333

**Published:** 2020-06-25

**Authors:** Jongnam Hwang, Giang Thu Vu, Bach Xuan Tran, Thu Hong Thi Nguyen, Bang Van Nguyen, Long Hoang Nguyen, Huong Lan Thi Nguyen, Carl A. Latkin, Cyrus S. H. Ho, Roger C. M. Ho

**Affiliations:** 1 Division of Social Welfare and Health Administration, Wonkwang University, Iksan, Korea; 2 Center of Excellence in Evidence-based Medicine, Nguyen Tat Thanh University, Ho Chi Minh City, Vietnam; 3 Institute for Preventive Medicine and Public Health, Hanoi Medical University, Hanoi, Vietnam; 4 Bloomberg School of Public Health, Johns Hopkins University, Baltimore, United States of America; 5 Hanoi Heart Hospital, Hanoi, Vietnam; 6 Department of Hemato-Toxico-Radiology and Occupational Disease, Hospital 103, Military Medical University, Hanoi, Vietnam; 7 Institute for Global Health Innovations, Duy Tan University, Da Nang, Vietnam; 8 Department of Psychological Medicine, National University Hospital, Singapore, Singapore; 9 Department of Psychological Medicine, Yong Loo Lin School of Medicine, National University of Singapore, Singapore, Singapore; 10 Institute for Health Innovation and Technology (iHealthtech), National University of Singapore, Singapore, Singapore; University of Ghana College of Health Sciences, GHANA

## Abstract

Patient satisfaction is a useful predictor of adherence and outcomes of cardiovascular diseases (CVDs) treatment. This study explored the satisfaction of Vietnamese CVDs inpatients and outpatients using a scale specifically designed for CVDs patients and examined the factors associated with satisfaction towards CVDs treatment services. Interviews of 600 patients at the Hanoi Heart Hospital were conducted. We developed a measurement scale for both inpatient and outpatient services. Multivariate Tobit regression was used to determine the associated factors with patient satisfaction. For inpatients, Cronbach’s alpha reported for the domains were in the range of 0.72–0.97, while for outpatients, Cronbach’s alpha was within 0.61–0.97. Overall, patients were more satisfied with inpatient services (Mean = 81.8, SD = 5.8) than outpatient services (Mean = 79.7, SD = 5.2, p<0.05). In inpatients, the highest complete satisfaction was in “Attitude of Nurse” item (42.0%), the highest satisfaction score was in “Care and treatment” domain (Mean = 85.6, SD = 9.7) and the lowest in “Hospital facilities” domain (Mean = 78.3; SD = 9.2). Among outpatients, the highest complete satisfaction was in “Attitude of physicians when examining, guiding and explaining to the patient” item (19.7%), the highest satisfaction score was in “Attitude of medical staff” domain (Mean = 82.8; SD = 7.9) and the lowest in “Waiting time” domain (Mean = 76.6; SD = 8.2). People not having health insurances had significantly higher scores in “Waiting time”, “Hospital facilities” and “Attitude of staff” domains (for outpatients) and in “Health service accessibility”, “Hospital facilities” domains (for inpatients) as well as higher total satisfaction score than those having health insurance. Findings discovered through the application of the newly developed instrument showed low satisfaction regarding hospital facilities for inpatients and waiting time for outpatients, suggesting renovation efforts, while inferiority regarding patient satisfaction of health insurance covered patients compared to those without implied policy reform possibility. Further enhancement and validation of the developed instrument was required.

## Introduction

Patient satisfaction has long been considered a pivotal element not only for achieving an optimal relationship between patient and health professionals/providers but also for the design of quality assurance and improvement initiatives [1, 2]. From the perspective of healthcare service providers, assessment of patient's experience and satisfaction with health care services could capture whether the services attend to an acceptable standard and highlight potential areas for quality improvement [3]. In addition, although the correlation between patient satisfaction and positive health outcomes have not been found to be definitive [4], a number of studies indicated that satisfied patients were more likely to exert better treatment adherence, which potentially would lead to outcome improvement over time [5, 6]. Enhancing patient satisfaction has also been found to be an effective way to improve patient retention and referrals [7], consequently, increase the market presence and revenue of health facilities [5].

Cardiovascular diseases (CVDs) are among the most significant health problems of the 21^st^ century, causing not only more fatality than any other leading cause of deaths [8] but also substantial morbidity and disability [9]. The burden of CVDs on low and middle-income countries (LMICs) has been disproportionately higher than that on high-income countries [10, 11], partly due to under-developed health systems having limited capability in providing sufficient and effective CVDs care [12]. Cardiovascular conditions include both acute episodes and long term disability that require curative care as a component of a continuous coordinated care model covering primary care, hospital care and post-acute care [13]. Given the influence of patient satisfaction on quality and effectiveness of acute/ hospital care as well as the acute on chronic nature of CVDs, assessing patient satisfaction toward healthcare services among CVDs patients in LMICs setting can be seen as crucial to improve patient experience and health status of these patients.

Nonetheless, how to assess patient satisfaction has still been a subject of debate in literature for the last decades, with measuring patient satisfaction claimed to be a rather challenging [14]. One of the issue with patient satisfaction measurement is arguably the subjective and complex nature of ‘satisfaction’–how a patient define their satisfaction would be influenced by their expectations and grounded on their set of “values, beliefs, attitudes and experiences” [14]. Patient satisfaction measurement developed for and utilized on patients from one country, for instance, would probably face adaptation problems when being applied to people from another country, due to cultural variations and dissimilar characteristics of diverse health systems [15]. Another concern over patient satisfaction measurement has been the reliability and/ validity of measuring instruments [16]. Numerous tools have been developed to examine patient satisfaction, including ones provided by private companies and those of publicly and standardized ones such as consumer assessment health plans (CAHPS) [17] and patient satisfaction questionnaires (PSQ-18) [18], yet criticisms remain that although these existing instruments had good validity and reliability, the scope of these tools was limited such as difficulties in customization, not including professionalism, hospital environment or privacy and security [2]. On the other hand, internally developed instruments that aim to capture specific aspects of patient care, being created through entirely *de novo* generation or adoptions of questions from other prevailing standardized tools, have been proven to be applicable in cases when the existing measuring scales deemed inappropriate [3].

Thus, an attempt to examine the patient satisfaction of people suffering CVDs, especially in low resource settings would first require the construction and evaluation of a contextualized patient satisfaction scale. Such exercise is particularly important in the context of Vietnam, given the high prevalence and burden of CVDs in the country as CVDs accounted for 31% of all deaths in Vietnam in 2016 [19] and the lack of literature exploring the issue of patient satisfaction among CVD patients. Furthermore, the establishment of an instrument with appropriate validity would potentially facilitate comparison in a wider context, for instance between different medical settings or diseases. This study aimed to investigate the patient satisfaction towards hospital care of people with CVDs, for both inpatient and outpatient services, by employing a scale specifically designed for the study. The reliability and validity of such an instrument would be examined prior to application. In addition, factors potentially associated with CVDs patients’ satisfaction would also be explored.

## Materials and methods

### Study designs and participants

A cross-sectional study was conducted in Hanoi Heart Hospital, one of the largest central cardiovascular hospitals from July to December 2016. There were approximately 600 outpatients visiting the hospital per day. Moreover, this hospital had 276 inpatient beds with 276 inpatients being treated at the same time in the hospital. The patients admitted in this hospital were not only from Hanoi but also referred by health facilities of provincial and lower levels across Vietnam, thus were likely to reflect diverse socio-economic backgrounds well as disease profiles of Vietnamese heart patients. We invited both inpatients and outpatients who had been examined and treated in Hanoi Heart Hospital to participate in the study. The eligibility criteria: 1) Able to respond to the questionnaire (i.e. not suffering critical physical or psychological conditions that could affect responses); 2) Utilizing services in the hospital during the study period and 3) Accepting to participate in the study. We first briefly explain the study and then invited eligible patients to enroll in the study and asked them to provide written informed consent.

In this study, we used the formula to estimate a population proportion with specified relative precision to compute the necessary sample size. We used α = 0.05, anticipated population proportion P = 0.86 (based on a previous study on patient satisfaction in Vietnam [20]), relative precision ɛ = 0.05. The essential sample size was 251 patients per group. We added 20% for compensating drop-out patients given that most of the patients were older adults or had severe health states which might be more likely to refuse to participate or withdraw to the study. The sample consists of 600 patients (included 300 out-patients and 300 in-patients from 7 departments in Hanoi Heart Hospital) with the response rate of 100%.

### Measures and instruments

We conducted face-to-face interviews using a structured questionnaire. The data collection team included well-trained post-graduate students in the field of Public Health. The questionnaires were designed based on the Patient Satisfaction Questionnaire from the Ministry of Health [21]. The variables of concern were described as below:

### Patient satisfaction

**Fig 1:**
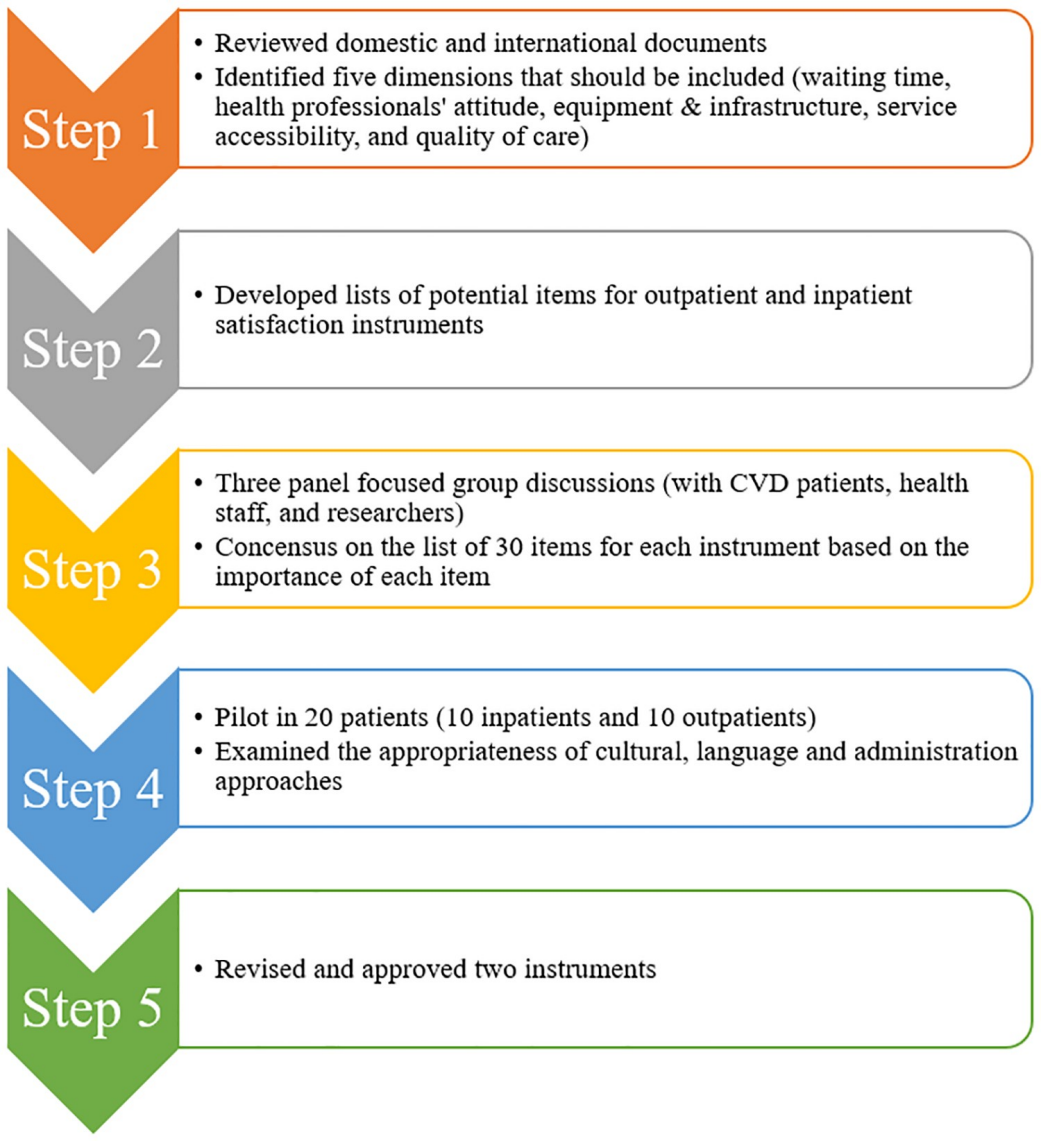
Tool development process.

A systematic procedure was used to develop a patient's satisfaction scale. First, we reviewed national and international literature to determine the potential items of patient satisfaction with heart-related services [22–24]. We identified following dimensions that should be included in the instrument: 1) Waiting time; 2) Attitude of physicians, nurses and other medical staffs; 3) Equipment and infrastructure of the hospital; 4) The accessibility of essential health service, and 5) Quality of care and treatment. However, because outpatients and inpatients have different procedures (e.g. outpatients might use more services than inpatient), we developed a separate list of items for outpatients and inpatients. Then, we performed three focused group discussions with patients with heart diseases, health staff, and researchers to examine the face validity of the instrument. Finally, we developed a list of items and selected them based on the importance of each item through the focus group discussions. We then piloted the tool in 20 patients (10 outpatients and 10 inpatients at the Hanoi Heart Hospital) to examine the appropriateness of cultural, language and administration approaches. After receiving patients’ feedback about the text, logical order of the questionnaire, we revised and finalized the questionnaire, as well as sought the approval from leaderboard of the hospital for survey. Finally, there were two questionnaires: one questionnaire measured satisfaction of outpatients, and another one measured satisfaction of inpatients. Each questionnaire had 30 items. The response options included a 5-level Likert scale from 1 “complete dissatisfaction” to 5 “complete satisfaction”, respectively. The score of each domain was calculated by summing the score of domain-related items and then transform to the 100-point scale, in which higher scores meant higher levels of satisfaction. The tool development process was illustrated in Fig 1.

### Other characteristics

We also collected socio-demographic characteristics, comprising age, living area, educational attainment, gender, and health insurance. Health-related quality of life (HRQOL) of patients were examined by using the EuroQol-5 Dimensions– 5 Levels (EQ-5D-5L). Five evaluated dimensions include mobility, self-care, usual activity, pain/discomfort and anxiety/depression. Each dimension has five options for response from no problem to extreme problem [25], producing 3125 possible health states, which can be converted to 3125 corresponding health utility index (or EQ-5D index) by using the Vietnamese cross-walk value set [26]. Patients answering “No problem” was classified into “No problem” group; otherwise, they were categorized into “Having problem” group. Furthermore, we used the EQ-Visual Analogue Scale (VAS) to measure the global health state of patients, with a score ranging from 0 “The worse health that you can imagine” to 100 “The best health that you can imagine” [25]. The EQ-VAS score was transformed to 1-point scale by dividing patients’ score to 100.

### Statistical analysis

Stata version 13 (Stata Corp. LP, College Station, USA) was used to analyze the data. Exploratory factor analysis (EFA) was used to determine construct validity of the scales by identifying factors and distributing items into these factors in order to enhance the scales’ interpretability. Eigenvalues of 1.30 (for outpatient satisfaction scale) and 1.20 (for inpatient satisfaction scale) were used to define a threshold to flatten out the eigenvalue curves. These thresholds were determined by using the scree test (in S1 and S2 Figs). In addition, Orthogonal Varimax rotation with Kaisers' normalization were applied to reconstruct the items. A value of 0.35 was used as the cut-off point for factor loadings. Cronbach’s alpha was computed to examine the internal consistency of scales and subscales. Because data on patient satisfaction were right censored (with 100 point as maximum score), multivariate Tobit regression was used to determine the associated factors with each domain of the scale. P-value <0.05 was considered the statistical significance.

### Ethics approval

This research proposal was approved by the IRB of Hanoi Medical University. Participants were requested to give written informed consent and were informed that they could withdraw at any time. Their contact details were coded and ensured to be confidential (code: 03.18/HDDDDHYHN).

## Results and discussion

Table 1 showed the demographic characteristics of respondents. The mean age of patients was 57.2 (SD = 19.9) years old. The majority was female (58.5%), having less than high school education (65.5%), living in rural areas (53.3%) and having health insurance (82.7%). There were statistically significant differences in gender, education, age and having health insurance between outpatients and hospitalized patients in this study.

**Table 1:** Demographic characteristics of respondents.

Characteristics	Outpatients		Inpatients		Total		p-value
	n	%	n	%	n	%	
**Total**	300	50.0	300	50.0	600	100.0	
**Gender**							
Male	89	29.7	160	53.3	249	41.5	<0.01
Female	211	70.3	140	46.7	351	58.5	
**Education**							
< High school	189	63.0	204	68.0	393	65.5	<0.01
High school	71	23.7	39	13.0	110	18.3	
> High school	40	13.3	57	19.0	97	16.2	
**Living location**							
Urban	141	47.0	139	46.3	280	46.7	0.87
Rural	159	53.0	161	53.7	320	53.3	
**Having health insurance**							
Yes	212	70.7	284	94.7	496	82.7	<0.01
No	88	29.3	16	5.3	104	17.3	
**Having problems in**							
Mobility	69	23.0	80	26.7	149	24.8	0.30
Self-care	46	15.3	73	24.3	119	19.8	<0.01
Usual activities	58	19.3	78	26.0	136	22.7	0.51
Pain/ Discomfort	129	43.0	104	34.7	233	38.8	0.04
Anxiety/Depression	124	41.3	87	29.0	211	35.2	0.02
	**Mean**	**SD**	**Mean**	**SD**	**Mean**	**SD**	
**Age**	59.5	12.9	54.8	24.9	57.2	19.9	<0.01
**EQ-5D index**	0.82	0.20	0.81	0.22	0.82	0.21	0.76
**EQ-VAS**	0.77	0.13	0.79	0.14	0.78	0.14	0.02

Table 2 revealed that after conducting EFA for the patient satisfaction scale for inpatient service, five domains were detected namely: “Attitude of medical staff” (14 items), “Care and Treatment” (4 items), “Health service accessibility” (5 items), “Waiting time” (4 items) and “Hospital facilities” (3 items), with the values of Cronbach’s alpha being 0.97; 0.94; 0.79; 0.72; and 0.74, respectively. The highest percentage of people having complete satisfaction was 42.0% in “Attitude of Nurse”, while the lowest percentage was 4.3% in “Flat hospital walkway and corridor, which made people easy to go”. People had the highest score in “Care and treatment” (Mean = 85.6, SD = 9.7) and the lowest score in “Hospital facilities” (Mean = 78.3; SD = 9.2).

**Table 2:** Factor loading and reliability of the patient satisfaction scale for inpatient service.

	% completely satisfy	The attitude of medical staff	Care and treatment	Health service accessibility	Waiting time	Hospital facilities
Nurses guide patients on how to take medicine daily	27.7	0.868				
Medical staff informed about the drug clearly to patients before taking drugs	27.3	0.866				
An attitude of physicians and nurses when doing professional techniques on patients	31.7	0.852				
Staff guide carefully administrative procedure when the patient discharge	24	0.849				
Physicians guide carefully patients how to self-care	30.3	0.833				
Attitude of physicians	40.3	0.814				
Physicians explain carefully illness conditions to the patient	34.7	0.81				
Attitude of nurses	42	0.801				
An attitude of medical staff when patients admitted to the hospital	40.3	0.795				
The attitude of staff when doing payment procedures	21.7	0.78				
Provide sufficient hospital clothes	26	0.755				
Physicians guide carefully when the patient discharge	25	0.752				
Medical staff had a friendly attitude when communicating	42	0.703				
Hospital has good security	21.3	0.601				
Satisfaction with the overall effectiveness of service	28		0.876			
Physicians’ quality of diagnosis and treatment	28		0.85			
Satisfaction with overall quality of care	33.3		0.78			
Nurse’s quality of care and treatment	37.3		0.704			
Clear and understandable signboards, maps to show the way to go to the Department,	5.7			0.816		
Building block, stair, rooms are easy to find	5.3			0.805		
Time for visiting patients is informed clearly	4.7			0.686		
Patients can call or ask medical staff if need.	19.7			0.685		
Flat hospital walkway and corridor, which made people easy to go	4.3			0.581		
Waiting time for diagnosis	11				0.724	
Waiting time for admitting hospital	10				0.699	
Waiting time when needing medical staff	13.3				0.6	
Waiting time when paying hospital fee	19.6				0.564	
Equipment and facilities in the room are sufficient	7.3					0.824
Equipment and facilities for doing professional techniques are sufficient	7.7					0.765
Hospital had the good hygienic condition	10					0.686
Cronbach’s Alpha		0.966	0.940	0.794	0.724	0.736
Mean		85.5	85.6	80.1	79.5	78.3
SD		8.6	9.7	6.0	7.3	9.2

Table 3 showed the EFA results for the patient satisfaction scale for outpatient service. We also separated these items into five domains namely: “Attitude of medical staff” (10 items), “Care and Treatment” (3 items), “Health service accessibility” (4 items), “Waiting time” (10 items) and “Hospital facilities” (3 items), with the values of Cronbach’s alpha being 0.97; 0.86; 0.90; 0.91; and 0.61, respectively. The highest percentage of people having complete satisfaction was 19.7% in “Attitude of physicians when examining, guiding and explaining to the patient”, while the lowest percentage was 4.3% in “Good hygienic condition”. People had the highest score in “Attitude of medical staff” (Mean = 82.8; SD = 7.9) and the lowest score in “Waiting time” (Mean = 76.6; SD = 8.2).

**Table 3:** Factor loading and reliability of the patient satisfaction scale for outpatient service.

	% completely satisfy	The attitude of medical staff	Care and treatment	Health service accessibility	Waiting time	Hospital facilities
The attitude of the staff in the abdominal/ vascular ultrasound department	17	0.942				
The attitude of staff in the echocardiography department	17.7	0.941				
The attitude of staff at the laboratory	17.3	0.941				
The attitude of staff in electrocardiogram room	17.3	0.933				
The attitude of staff collecting hospital fee	18.3	0.901				
The attitude of the nurse to patients before the examination	18.3	0.881				
Attitude of receptionist	18	0.877				
The attitude of physicians when examining, guiding and explaining to the patient	19.7	0.815				
Security staff had the good attitude with patients	19.3	0.806				
Hospital had good security condition	11.7	0.59				
Satisfaction with the overall effectiveness of service	11.3		0.872			
Physicians’ quality of diagnosis and treatment	10.3		0.86			
Satisfaction with overall quality of care	12.7		0.676			
Building block, stair, rooms are easy to find	5.3			0.906		
Clear and understandable signboards, maps to show the way to go to the Department,	5.3			0.895		
Flat hospital walkway and corridor, easy to go	5.3			0.85		
Patients can call or ask medical staff if need.	10.7			0.73		
Waiting time for X-ray and getting X-ray results	5				0.825	
Waiting time for payment procedure at the reception desk	7				0.810	
Waiting time for electrocardiogram	6.3				0.797	
Waiting time for abdominal/vascular ultrasound	5				0.786	
Waiting time for counseling	7				0.785	
Waiting time for echocardiography	5				0.763	
Waiting time for a medical examination	6				0.743	
Waiting time for testing and getting test results	5.7				0.682	
Waiting time for taking medicine at the drug store	7.7				0.644	
Waiting time for reading the results at physician’s room	5				0.632	
Good hygienic condition	4.3					0.779
Ensuring the confidentiality and privacy when examination or doing technique	6					0.691
Good light and water	4.7					0.589
Cronbach’s Alpha		0.973	0.863	0.900	0.913	0.612
Mean		82.8	80.5	80.4	76.6	78.1
SD		7.9	8.0	5.9	8.2	7.5

Table 4 compared the score of different domains of patient satisfaction according to EFA results. Domains "Attitude of staff", "Care and treatment", and "Hospital facilities" had significantly higher scores in inpatients than those in outpatients (p<0.01). Meanwhile, there was no difference in "Health service accessibility" and "Hospital facilities" for inpatients and outpatients. Overall, patients were more satisfied with inpatient service (Mean = 81.8, SD = 5.8) than outpatient services (Mean = 79.7, SD = 5.2, p<0.01).

**Table 4:** Comparison of patient’s satisfaction between inpatients and outpatients.

Factors	Inpatients			Outpatients			p-value
	Mean	SD	% rate 100 point	Mean	SD	% rate 100 point	
Health service accessibility	80.1	6.0	5.0	80.4	5.9	3.3	0.60
Waiting time	79.5	7.3	4.3	76.6	8.2	4.3	<0.01
Hospital facilities	78.3	9.2	9.7	78.1	7.5	6.3	0.82
Attitude of staff	85.5	8.6	4.0	82.8	7.9	14.3	<0.01
Care and treatment	85.6	9.7	8.0	80.5	8.0	26.0	<0.01
**Total score**	**81.8**	**5.8**	**1.7**	**79.7**	**5.2**	**1.3**	**<0.01**

Table 5 presents the results of multivariate regression to identify the predictors of the satisfaction score among outpatients. People not having health insurance had significantly higher scores in “Health service accessibility”, “Waiting time”, “Hospital facilities” and “Attitude of staff” domains as well as “Overall satisfaction”. People having pain/discomfort, anxiety/depression and problems with self-care had significantly lower satisfaction scores in “Care and Treatment” and overall satisfaction. Similarly, having a higher EQ-5D index was associated with a significantly lower satisfaction score in similar domains.

**Table 5:** Factors associated with patient satisfaction among outpatients.

Variables	Health service accessibility		Waiting time		Hospital facilities		Attitude of staff		Care and treatment		Overall satisfaction	
	Coef.	95%CI	Coef.	95%CI	Coef.	95%CI	Coef.	95%CI	Coef.	95%CI	Coef.	95%CI
**Age groups (vs < 30 years**^a^**)**												
30-<45 years	1.7	-3.8; 7.1	5.4	-1.9; 12.7	-1.7	-8.4; 4.9	-9.7*	-17.4; -2.0	-1.9	-9.4; 5.5	-1.0	-5.6; 3.5
45-<60 years	1.9	-3.2; 7.1	5.1	-1.7; 12.0	0.4	-5.9; 6.6	-7.0	-14.3; 0.3	-0.1	-7.1; 6.8	0.1	-4.1; 4.4
≥ 60 years	1.5	-3.6; 6.6	4.0	-2.8; 10.7	-1.1	-7.2; 5.1	-6.6	-13.8; 0.7	-1.2	-8.1; 5.7	-0.5	-4.7; 3.7
**Gender (Female vs Male**^a^**)**	0.8	-0.8; 2.4	1.5	-0.7; 3.6	0.1	-1.9; 2.0	0.5	-1.8; 2.7	0.4	-1.8; 2.6	0.6	-0.7; 2.0
**Education attainment (vs < High school**^a^**)**												
High school	-1.0	-2.7; 0.8	-1.2	-3.6; 1.1	-2.0	-4.1; 0.2	-0.1	-2.5; 2.3	-0.6	-3.0; 1.7	-0.9	-2.4; 0.5
> High school	-1.2	-3.4; 1.1	1.6	-1.4; 4.6	-1.3	-4.0; 1.5	1.6	-1.5; 4.8	-1.2	-4.2; 1.8	-0.1	-2.0; 1.7
**Living location (Rural vs Urban**^a^**)**	-0.5	-2.0; 1.0	-0.6	-2.6; 1.4	1.9*	0.1; 3.7	-0.1	-2.2; 1.9	-1.4	-3.4; 0.6	-0.1	-1.4; 1.1
**Having health insurance (No vs Yes**^a^**)**	1.6*	0.0; 3.2	3.2*	1.1; 5.3	3.0*	1.0; 4.9	2.7*	0.5; 4.9	0.3	-1.9; 2.4	2.0*	0.7; 3.3
**Having problems in**												
Pain/ Discomfort (Yes vs No^a^)	-2.5	-6.3; 1.2	-3.6	-8.6; 1.4	-3.3	-7.9; 1.2	-4.8	-9.9; 0.4	-9.9*	-15.2; -4.6	-4.4*	-7.4; -1.3
Anxiety/Depression (Yes vs No^a^)	-0.3	-3.3; 2.6	-3.8	-7.8; 0.1	-2.1	-5.7; 1.4	-1.6	-5.6; 2.5	-5.7*	-9.9; -1.6	-2.5*	-4.9; -0.0
Mobility (Yes vs No^a^)	-1.9	-5.3; 1.6	1.8	-2.9; 6.4	-0.9	-5.1; 3.4	-0.7	-5.5; 4.1	-1.3	-6.1; 3.5	-0.5	-3.4; 2.4
Self-care (Yes vs No^a^)	-0.8	-5.2; 3.6	-3.0	-8.9; 2.8	2.3	-3.0; 7.7	-2.4	-8.4; 3.6	-7.7*	-13.7; -1.7	-2.0	-5.7; 1.6
Usual activities (Yes vs No^a^)	1.0	-3.4; 5.4	-1.1	-7.0; 4.8	-3.9	-9.3; 1.4	-0.4	-6.5; 5.6	-3.2	-9.3; 2.8	-1.5	-5.1; 2.2
**EQ-VAS**	-1.8	-7.8; 4.1	4.0	-3.9; 12.0	5.9	-1.3; 13.1	1.7	-6.5; 9.9	1.7	-6.4; 9.7	2.3	-2.6; 7.2
**EQ-5D index**	-7.4	-26.3; 11.6	-16.2	-41.5; 9.1	-17.8	-40.8; 5.3	-15.0	-41.2; 11.1	-44.8*	-72.2; -17.5	-18.2*	-33.9; -2.5

^a^ reference group;

*p<0.05.

Table 6 presents the factors associated with patient satisfaction among inpatients. Being older than 60 years old was found to associated with significantly higher satisfaction in “Waiting time”, “Hospital facilities”, “Care and treatment” domains and overall satisfaction. Females had significantly higher scores in “Health service accessibility”, “Hospital facilities”, “Attitude of staff” domains as well as overall. Having anxiety/depression was found to correlate with the lower score in the domains of “Waiting time”, “Attitude of staffs” and Overall satisfaction. In contrast, people having problems with self-care reported significantly higher satisfaction score overall as well as in “Health service accessibility” and “Waiting time” domains. People not having health insurance had higher scores in “Health service accessibility”, “Hospital facilities” and overall satisfaction.

**Table 6:** Factors associated with patient satisfaction among inpatients.

Variables	Health service accessibility		Waiting time		Hospital facilities		Attitude of staff		Care and treatment		Overall satisfaction	
	Coef.	95%CI	Coef.	95%CI	Coef.	95%CI	Coef.	95%CI	Coef.	95%CI	Coef.	95%CI
**Age groups (vs < 30 years**^a^**)**												
30-<45 years	-0.7	-3.8; 2.5	0.2	-3.5; 4.0	-0.6	-5.5; 4.3	2.8	-2.2; 7.7	-0.4	-7.0; 6.2	0.1	-2.8; 2.9
45-<60 years	-1.0	-3.4; 1.5	1.4	-1.5; 4.3	-0.0	-3.8; 3.7	-0.2	-4.0; 3.5	2.0	-3.1; 7.1	0.2	-2.0; 2.4
≥ 60 years	0.1	-2.0; 2.2	2.5	-0.0; 5.0	3.4*	0.1; 6.6	2.6	-0.7; 5.9	5.2*	0.8; 9.7	2.3*	0.4; 4.2
**Gender (Female vs Male**^a^**)**	1.6*	0.2; 3.0	0.9	-0.8; 2.5	2.0	-0.2; 4.2	2.7*	0.5; 4.9	2.1	-0.9; 5.1	1.6*	0.3; 2.9
**Education attainment (vs< High school**^a^**)**												
High school	-1.1	-3.2; 1.1	0.5	-2.1; 3.1	2.3	-1.1; 5.7	0.0	-3.3; 3.4	1.3	-3.3; 5.8	0.4	-1.6; 2.4
> High school	0.3	-1.6; 2.3	1.6	-0.7; 3.9	-1.1	-4.1; 2.0	0.5	-2.6; 3.5	0.2	-3.9; 4.4	0.1	-1.6; 1.9
**Living location (Rural vs Urban**^a^**)**	-0.3	-1.8; 1.2	-0.3	-2.1; 1.5	-1.8	-4.1; 0.6	-1.4	-3.7; 1.0	-1.3	-4.5; 1.8	-0.9	-2.2; 0.5
**Having health insurance (No vs Yes**^a^**)**	3.8*	0.6; 7.0	2.4	-1.3; 6.2	6.0*	1.0; 10.9	1.7	-3.2; 6.6	2.3	-4.3; 9.0	3.1*	0.2; 5.9
**Having problems in**												
Pain/Discomfort (Yes vs No^a^)	-1.7	-4.8; 1.3	-2.0	-5.6; 1.6	-2.6	-7.3; 2.1	-2.1	-6.8; 2.7	-0.6	-6.9; 5.8	-1.7	-4.4; 1.1
Anxiety/Depression (Yes vs No^a^)	-1.2	-3.6; 1.2	-3.9*	-6.7; -1.1	-0.9	-4.5; 2.8	-4.7*	-8.5; -1.0	-3.7	-8.7; 1.3	-2.5*	-4.7; -0.4
Mobility (Yes vs No^a^)	-0.1	-3.9; 3.7	2.2	-2.4; 6.7	-1.9	-7.8; 4.0	2.0	-3.9; 8.0	1.3	-6.9; 9.4	0.6	-2.9; 4.0
Self-care (Yes vs No^a^)	4.7*	0.8; 8.5	4.6	-0.0; 9.2	2.6	-3.4; 8.5	4.7	-1.3; 10.6	2.0	-6.2; 10.1	3.5*	0.1; 7.0
Usual activities (Yes vs No^a^)	-5.1*	-9.5; -0.7	-3.9	-9.1; 1.3	-0.3	-7.1; 6.4	-6.6	-13.4; 0.1	-2.6	-11.9; 6.6	-3.5	-7.4; 0.5
**EQ-VAS**	0.6	-5.3; 6.5	3.0	-4.1; 10.0	7.8	-1.3; 16.9	-8.9	-18.0; 0.3	-5.0	-17.4; 7.5	-0.1	-5.5; 5.2
**EQ-5D index**	-4.3	-18.8; 10.2	-2.8	-20.2; 14.5	-5.0	-27.5; 17.5	-0.8	-23.4; 21.8	2.7	-27.8; 33.2	-2.1	-15.3; 11.0

^a^ reference group;

*p<0.05.

## Discussion

In the absence of an officially available instrument to examine the satisfaction of patients with CVDs in Vietnam, we have developed and applied our own assessment questionnaire based on research on existing literature on the subject. With satisfactory internal consistency, our instrument can be used with suitable modifications to measure the level of satisfaction of patients with CVDs in various settings. The results of our analysis provide some interesting insights into the level and determinants of satisfaction among those with heart disease, which in turn suggest that improvements at both facility-level and policy-level are needed for improving outcomes of treatment services.

The Cronbach’s alpha reliability coefficient of this scale, as derived from our analyses, was within a range of values considered sufficient for a patient satisfaction scale to be reliable [27]. In general, test developers typically strive for an instrument with a coefficient for reliability in the range of .80 to .90, in some other cases a minimum of 0.7 for Cronbach’s alpha might be considered as a good result [27–29]. Our efforts in distinguishing between inpatient and outpatient services when developing the scale have enabled a more sophisticated capture of patient satisfaction regarding each of these services. Nonetheless, as the capacity of Cronbach’s alpha in determining the reliability of an instrument has been subjected to debate in existing literature [30, 31], it is recommended that future researches employing our scale conduct an additional investigation as well as consider alternative measures of instrument reliability.

In accordance with findings from other studies on patients’ perception of the quality of care, “Attitude of medical staff” and “Care and treatment” domains had the highest score in outpatient and inpatient groups, respectively [15]. A previous study indicated that positive experience with medical staffs is closely associated with the subjective evaluation of the patients regarding quality of health care received during their hospital stay [28], while other researches highlighted the importance of having confidence in expertise and attentiveness of nurses and doctors on perception of patients on care quality [32, 33]. Existing literature also suggested that healthcare provider’s interpersonal communication skills and behaviors towards the patients were directly linked with patient satisfaction [34] This implies the dependency of providing the better quality of care on enhancing the capacity of medical staff both in terms of expertise and interpersonal, “soft” skills. Meanwhile, “Hospital facilities” appeared to be the area that more attention should be focused in an attempt to improve the satisfaction of inpatients, in line with findings elsewhere [35]. It has been proposed that since patients cannot reliably differentiate positive experiences with the physical environment from positive experiences with care, an improved, patient-centered hospital environment with features like reduced noise, improved natural light, visitor-friendly facilities, well-decorated rooms, and hotel-like amenities would lead to higher satisfaction of patients as well as medical staff [36]. The need for renovating and upgrading the physical environment of the hospitals thus should not be overlooked. "Waiting time" was the lowest score for outpatients, reflecting a well-established argument that longer waiting periods were negatively correlated with patient satisfaction ratings from the healthcare provider [37]. As a result, consideration should be made in conducting systematic metrics to reduce a long wait-times at the hospital level as part of the provision of better quality services [38].

In addition, our results echo findings from a number of existing studies where older age was reported to be positively associated with satisfaction score among those using inpatients services [39–41]. On the other hand, the correlation between being female and higher satisfaction scores found in our study adds to the wealth of mixed evidence on relationship between gender and satisfaction: while some studies found male gender to be the predictor of higher patient satisfaction score [39–41], others found opposite results [42] or no significant association [43]. Nonetheless, it was interesting to note that compared with those having health insurance, people without health insurance had a higher score in overall satisfaction as well as in domains relating to care accessibility and hospital facilities. This partly reflected an issue suggested elsewhere regarding the implementation of health insurance in Vietnam context, when patients covered by health insurance faced a higher likelihood of receiving a lower quality of services compared to those who make out-of-pocket, full payment for services instead of utilizing health insurance or due to lack of health insurance [44]. The existence of premium services at hospital–providing patients with higher quality care and facilities, a private room with nurses on call at all time, for instance [45], which are not covered by insurance may also be a reason for higher perceived satisfaction among those paying out-of-pocket in comparison with those using insurance-covered services. In addition, the lower satisfaction score given for “Attitude of staff” of those having insurance may be a sign of a problem that has been made aware by medical authority and reported by the media–the discrimination of health staff towards insurance holders [46, 47]. Nonetheless, further investigation into this matter should be encouraged to improve the effectiveness of the health insurance system and ensure fairness in the treatment received by patients.

This study has several implications. As our newly developed contextualized patient satisfaction scale was proved to be internally reliable, it can be utilized as the basis for further enhancement and validation that would ideally involve the support of authorities–Ministry of Health, for instance, to be sufficiently reliable for application beyond one hospital setting. Insights regarding the perceived satisfaction of patients with heart disease discovered through the application of such scale would pinpoint directions for improving the quality of care and experience of patients with treatment. This would be immensely valuable for Vietnamese hospitals as managerial and financial autonomy introduced through hospital administration reform imposed by the government force them to strive for better performance as service providers. Measuring the satisfaction of patients can also be conducted periodically as part of a quality assurance process as well as a component of larger service improvement strategies. In addition, finding concerning generally lower satisfaction of health insurance covered patients over non-covered ones prompts examination and possible reform of health insurance policy.

Nevertheless, the findings of this study should be view in light of its inherent limitations. First, the cross-sectional of the survey would allow only a ‘snap-shot' capture of the current situation. The self-reported design of the questionnaire meant that answers would be subjective. In addition, although the attempt was made to include a reasonably large number of participants in this study, further researches involving large sample sets and/or several studied sites are called for to better understand the factors associated with patient satisfaction and potentially enhance the design of the scale. Future studies should also consider clustering of patients based on their response, as such analysis would potentially shed more lights on heterogeneity and other sub-structures in patients’ experiences, providing a better understanding of patient satisfaction and associated factors.

## Conclusions

The aim of this research is to evaluate the cardiovascular patient’s satisfaction in Vietnam through a patient satisfaction measurement scale developed and validated especially for the concerned population. Analyses indicated the scale to be internally reliable. Findings discovered through the application of the newly developed instrument showed low satisfaction regarding hospital facilities for inpatients and waiting time for outpatients, suggesting renovation efforts, while inferiority regarding patient satisfaction of health insurance covered patients compared to those without implied policy reform possibility. Further enhancement and validation of the developed instrument was required, which called for more support from the Ministry of Health.

## Supporting information

S1 Fig Scree plot for outpatient satisfaction scale. (TIF)

S2 Fig Scree plot for inpatient satisfaction scale. (TIF)
