# Supplementary figures and images for: Measuring satisfaction with health care services for Vietnamese patients with cardiovascular diseases
Source: PLoS One. 2020 Jun 25;15(6):e0235333. doi: 10.1371/journal.pone.0235333 (PMC7316281; doi:10.1371/journal.pone.0235333)

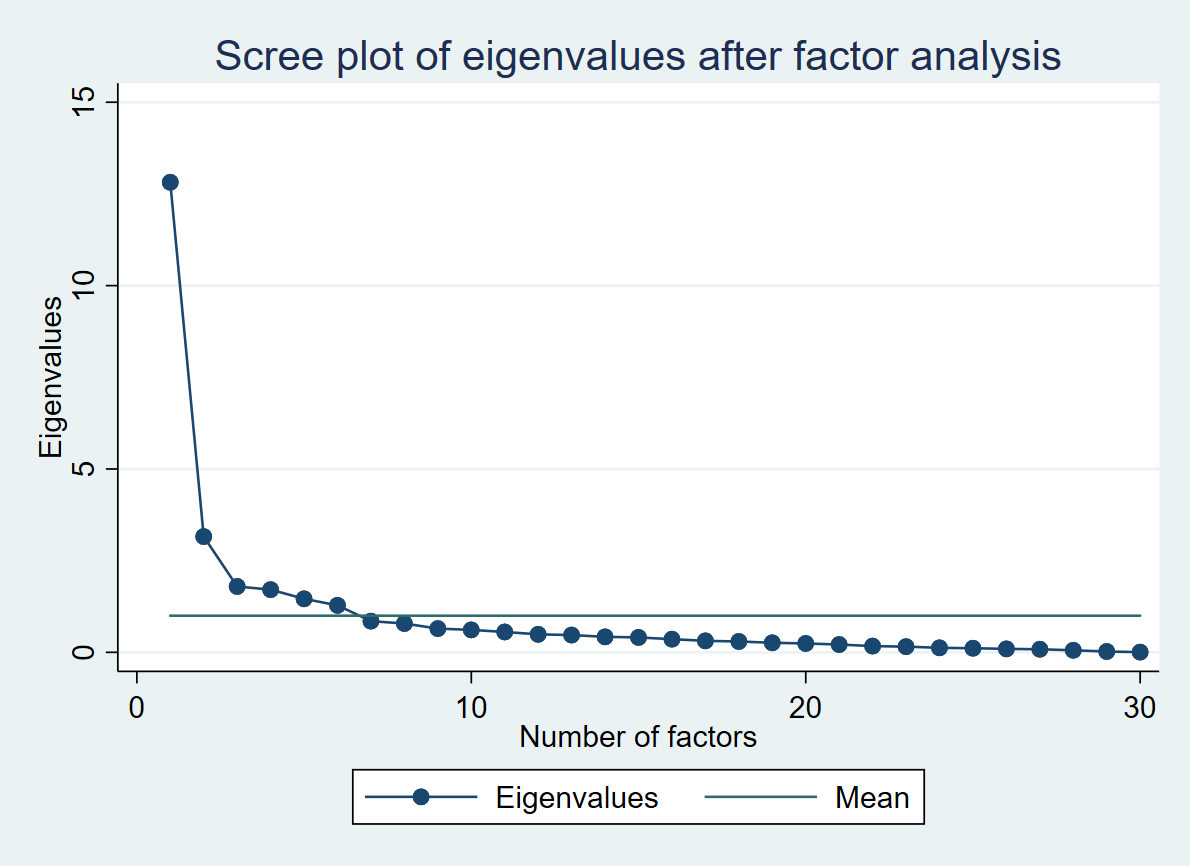

Supplement: S1 Fig — (TIF) [file pone.0235333.s001.tif]

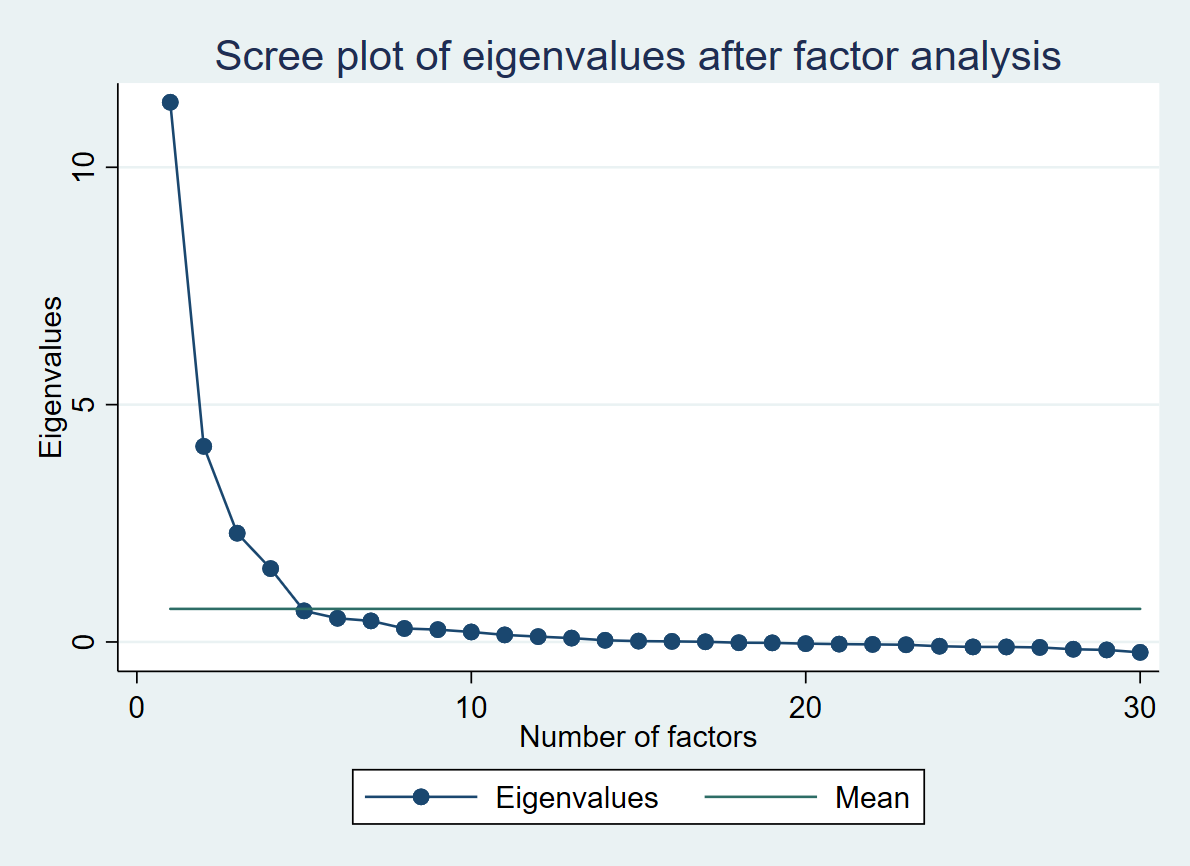

Supplement: S2 Fig — (TIF) [file pone.0235333.s002.tif]
